# 

**Published:** 1832-10-01

**Authors:** 


### CONTENTS

OF THE

### MEDICO-CHIRURGICAL REVIEW

No. XXXIV. OCTOBER 1, 1832.

### REVIEWS.

I.

Practical Observations in Midwifery. Part the Second. By Dr. RAMSBOTHAM .. 289

II.

Observations on the Healthy and Diseased Properties of the Blood. By Dr. STEVENS .. .. .. .. .. .. .. .. .. .. .. .. .. .. .. .. .. .. 321

IV.

A Practical Treatise on Uterine Hæmorrhage. By Mr. INGLEBY .. .. .. .. 337

V.

M. CAZENAVE on Diseases of the Skin .. .. .. .. .. .. .. .. .. .. .. .. 360

VI.

Dr. WEBSTER on Epidemic Cholera .. .. .. .. .. .. .. .. .. .. .. 383

VII.

Dr. REID on Medical Botany .. .. .. .. .. .. .. .. .. .. .. .. .. 387

VIII.

On the Forms, Causes, Sanability, and Treatment of Pulmonary Consumption. By Dr. BLACKMORE.. .. .. .. .. .. .. .. .. .. .. .. .. .. .. .. 430

IX.

Madras Medical Reports. By Mr. FLEMING.. .. .. .. .. .. .. .. .. .. 408

1. On Cholera .. .. .. .. .. .. .. .. .. .. .. .. .. .. .. .. 408
2. On Dysentery .. .. .. .. .. .. .. .. .. .. .. .. .. .. .. 409
3. On Sulphate of Quinine .. .. .. .. .. .. .. .. .. .. .. .. 409
4. On Phlebitis .. .. .. .. .. .. .. .. .. .. .. .. .. .. .. 410
5. Syphilis treated without Mercury .. .. .. .. .. .. .. .. .. .. 410

X.

Mr. JAMES on Inflammation .. .. .. .. .. .. .. .. .. .. .. .. .. 411

XI.

Dr. STEVENS on the Blood—(continued from page 336) .. .. .. .. .. .. .. 422

XII.

Dr. OTTO's Compendium of Human and Comparative Pathological Anatomy .. 427

XIII.

Mr. TOD on the Anatomy and Physiology of the Organ of Hearing .. .. .. .. 431

### PERISCOPE.

1. On fetid Abscesses in the Neighbourhood of Mucous Membranes .. .. .. 433
2. Aphonia treated by the Nitrate of Silver .. .. .. .. .. .. .. .. .. .. 435
3. Rheumatism treated by Acetate of Morphia locally .. .. .. .. .. .. 436
4. Sciatica cured by Bloodletting.. .. .. .. .. .. .. .. .. .. .. .. .. 436
5. Paralysis of the Portio Dura cured by Moxas.. .. .. .. .. .. .. .. 437
6. Epilepsy, with scrofulous Disease of the Cerebellum .. .. .. .. .. .. 438
7. Epidemic Disease among Poultry .. .. .. .. .. .. .. .. .. .. .. 439
8. Diabetes Mellitus .. .. .. .. .. .. .. .. .. .. .. .. .. .. .. 439
9. Scrofulous Disease of the Bones .. .. .. .. .. .. .. .. .. .. .. 439
10. Puerperal Peritonitis, followed by Perforation of the Abdominal Parietes .. 441
11. On some obscure Forms of Phlegmasiæ.. .. .. .. .. .. .. .. .. .. 442
12. Feeble Treatment of Typhus by the French.. .. .. .. .. .. .. .. 444
13. M. Roux on the Use of large flat Ligatures .. .. .. .. .. .. .. .. 444
14. Recent and infamous Official Mandate in France, on the Subject of the Wounded.. .. .. .. .. .. .. .. .. .. .. .. .. .. .. .. .. .. 445
15. Epidemic Miliary Sweating Fever in the Department of Oise .. .. .. .. 446
16. Anatomy and Physiology of the Optic Nerve .. .. .. .. .. .. .. .. 447
17. Report of the Royal Academy of France on Cholera .. .. .. .. .. .. 448
18. Memoir of the last Illness of Cuvier .. .. .. .. .. .. .. .. .. .. 451
19. Emphysema of the Lungs.. .. .. .. .. .. .. .. .. .. .. .. .. .. 453
20. Dr. Lee on the Structure of the Human Placenta .. .. .. .. .. .. 454
21. Observations on various Diseases. By Dr. Graves .. .. .. .. .. .. 458
  1. Ptyalism .. .. .. .. .. .. .. .. .. .. .. .. .. .. .. 458
  2. Cold Affusion in Convulsions.. .. .. .. .. .. .. .. .. .. .. 459
  3. Neuralgia of the Mammæ .. .. .. .. .. .. .. .. .. .. .. 461
  4. External Application of Narcotics.. .. .. .. .. .. .. .. .. 462
22. Angina Pectoris and Diseased Heart .. .. .. .. .. .. .. .. .. .. 463
23. Dr. Ferguson on Contagion .. .. .. .. .. .. .. .. .. .. .. .. 465
24. Sir D. Brewster on Ocular and Acoustic Illusions.. .. .. .. .. .. .. 467
  *a.* Various Ocular Illusions.. .. .. .. .. .. .. .. .. .. .. .. 469
  *b.* Accidental Colours, with Sir I. Newton's Case .. .. .. .. .. .. 471
  *c.* Insensibility to certain Colours .. .. .. .. .. .. .. .. .. .. 472
  *d.* Spectral Illusions .. .. .. .. .. .. .. .. .. .. .. .. .. 473
  *e.* Case of Mrs A. .. .. .. .. .. .. .. .. .. .. .. .. .. .. 474
  *f.* Remarks of Sir D. Brewster on Spectral Illusions .. .. .. .. .. 474
  *g.* Criticisms on the above .. .. .. .. .. .. .. .. .. .. .. .. 477
  *h.* Another Optical Illusion.. .. .. .. .. .. .. .. .. .. .. .. 478
  *i.* Acoustic Illusions .. .. .. .. .. .. .. .. .. .. .. .. .. 479
  *j.* Effects of great Elevation, &c. .. .. .. .. .. .. .. .. .. .. 480
  *k.* Remarkable Feats of Strength .. .. .. .. .. .. .. .. .. .. 481
  *l.* Capability of resisting great Heat.. .. .. .. .. .. .. .. .. .. 482
25. Death from Injection of an Encysted Dropsy .. .. .. .. .. .. .. .. 483
26. Fungous Tumours of the Dura Mater and Skull .. .. .. .. .. .. .. 483
27. Composition of Cow's Milk before and after Parturition .. .. .. .. .. 485
28. Mr. Rodweiss on the Decomposition of Uric by Nitric Acid .. .. .. .. 486
29. Vitreous Humor regenerated .. .. .. .. .. .. .. .. .. .. .. .. 486

30. Vesico-vaginal and Recto-vaginal Fistula cured. Three Cases .. 486
31. Tic Douloureux of a Stump, for which part of the Median Nerve was excised, 489
32. Dr. Watson on Hæmaturia .. 491
33. Mr. Earle's Lectures on Burns .. 492
a. Low Power of resisting Heat .. 494
b. Degrees of Burns .. 494
c. Various Plans of Treatment .. 495
d. Internal Stimulants .. 497
e. Secondary Consequences of Burns .. 498
34. Carus's Tables of Comparative Anatomy .. 501
35. Mr. Arnott and the Medico-Chirurgical Reviewer .. 503
36. Mr. Crampton on Injuries of the Head .. 507
a. Remarks on Hospital Reports .. 507
b. Treatment of Compound Fracture of the Skull in Dublin .. 511
c. Cases of the same .. 512
37. Dr. Macfarlane's Clinical Reports .. 515
1. Lupus .. 516
2. Lumbar Abscess .. 519
38. Affections of the Head produced by Quinine .. 522
39. Diuretic Properties of Lichen Vulgaris .. 523
40. Cases of Abscess of the Liver .. 523
41. Bite from the Karrait Snake .. 525
42. Native Lithotomists in India .. 526
43. Report from St. George's Hospital .. 527
1. Operation of Lithotomy for the Removal of a Piece of Gum Catheter .. 528
Remarks by Mr. Brodie .. 528
2. Diffused Hydrocele of the Cord .. 529
3. Fatal Case of Constipation .. 530
4. Fatal Results from Amputation of Fingers .. 530
5. Cases of Laceration of the Urethra .. 533
6. Fractured Pelvis .. 535
7. Fractured Sternum .. 536
44. Letter from Mr. Thomas to Dr. Ryan .. 537
45. Mr. Elliott's Case of Injury of the Head, followed by Discharge of Pus from the Ear, and Recovery .. 538
46. Mr. Gamack's Case of Ruptured Bladder .. 540
47. Cases of Phlebitis, illustrating the good Effects of free Openings .. 541
48. Cholera .. 544
a. Various Remedies .. 545
b. Contagion in Canada and Edinburgh .. 545
c. Last Circular of the Board of Health .. 546
d. The peripatetic Cholera Commissioners .. 547
e. Riot at Manchester .. 548
f. Remarks of the Times and on the Times .. 548
Excerpta Choleralogica .. 550
1. Saline Injections into the Veins .. 550
2. Mr. Buchanan's Plan .. 550
3. Bandaging the Abdomen .. 551

4. Anodyne Paste for Cholera.. .. .. .. .. .. .. .. .. .. .. .. .. .. 551
5. Cold Water for Cholera .. .. .. .. .. .. .. .. .. .. .. .. .. .. 551
6. Narcotic Glysters.. .. .. .. .. .. .. .. .. .. .. .. .. .. .. 552
7. Muriate of Soda .. .. .. .. .. .. .. .. .. .. .. .. .. .. .. 552
8. Prohibition of all Drink .. .. .. .. .. .. .. .. .. .. .. .. .. 553
9. Dr. Gregory and the Bills of Mortality .. .. .. .. .. .. .. .. .. 553
10. Salines unsuccessful in Cholera .. .. .. .. .. .. .. .. .. .. .. 555
11. Dr. Lindsey's Mode of Treatment .. .. .. .. .. .. .. .. .. .. 555
12. Treatment at Leeds .. .. .. .. .. .. .. .. .. .. .. .. .. .. 556
13. Calomel and Opium .. .. .. .. .. .. .. .. .. .. .. .. .. .. 556
14. A logical Deduction .. .. .. .. .. .. .. .. .. .. .. .. .. .. 556
15. Cold Water for Cholera .. .. .. .. .. .. .. .. .. .. .. .. .. 556
16. M. M. Foville and Parchappe on Cholera.. .. .. .. .. .. .. .. 558
17. Circular of the Board .. .. .. .. .. .. .. .. .. .. .. .. .. .. 559
49. Menstruation in different Countries. From Dr. Davis's Obstetric Medicine, 560
60. The Editor's Remarks on Cholera .. .. .. .. .. .. .. .. .. .. .. 562
*a.* Effects of Locality, as shewn by several Examples in England .. .. 562
*b.* Cholera in Lunatic Asyla .. .. .. .. .. .. .. .. .. .. .. .. 563
*c.* Labours of Dr. Mackintosh .. .. .. .. .. .. .. .. .. .. .. 563
51. Mr. Schloss's Anatomical Models .. .. .. .. .. .. .. .. .. .. .. 564
52. Dr. Arnott's Hydrostatic Bed .. .. .. .. .. .. .. .. .. .. .. .. 564
53. The Anatomy Act .. .. .. .. .. .. .. .. .. .. .. .. .. .. .. 566
*a.* Dr. Somerville's Circulars .. .. .. .. .. .. .. .. .. .. .. 570
*b.* Remarks upon the Act .. .. .. .. .. .. .. .. .. .. .. .. .. 571
54. Undercliff, Isle of Wight .. .. .. .. .. .. .. .. .. .. .. .. .. 573
55. Provincial Medical and Surgical Association.. .. .. .. .. .. .. .. 574
56. Death of Dr. Allsop .. .. .. .. .. .. .. .. .. .. .. .. .. .. .. 574
Bibliographical Record .. .. .. .. .. .. .. .. .. .. .. .. .. .. .. 573

Extra-Limites.

Dr. Hackett's Letter in reply to Dr. Stevens .. .. .. .. .. .. .. .. .. .. 577

